# Field Investigation of Traffic Characteristics in Africa Based on an Integrated Dynamic Traffic Monitoring System

**DOI:** 10.3390/s26072039

**Published:** 2026-03-25

**Authors:** Zining Chen, Xiao Du, Yuheng Chen, Zeyu Zhang, Zhihao Bai, Zhongshi Pei, Junyan Yi

**Affiliations:** 1 China Road & Bridge Corporation, Beijing 100011, China; chenzining@crbc.com (Z.C.); doux@crbc.com (X.D.); 2 School of Transportation Science and Engineering, Harbin Institute of Technology, Harbin 150090, China; 20s032029@stu.hit.edu.cn (Y.C.); 24s132085@stu.hit.edu.cn (Z.Z.); zhihao-bai@stu.hit.edu.cn (Z.B.); hitpzs@hit.edu.cn (Z.P.)

**Keywords:** traffic load characterization, weigh-in-motion (WIM), multi-scale traffic monitoring, heavy truck overloading, African highways

## Abstract

**Highlights:**

**What is the main finding?**

- A multi-source sensing and weigh-in-motion system was developed to reveal the temporal characteristics of traffic loading and provide reliable field data for traffic load characterization on African highways.

**What is the implications of the main finding?**

- The proposed monitoring and characterization framework provided a transferable methodology for traffic load modeling and infrastructure design optimization across developing regions.

**Abstract:**

Reliable traffic load characterization remains a critical challenge in many African countries due to the lack of continuous field measurements. This study developed an integrated dynamic traffic monitoring and weigh-in-motion system on representative highways in Kenya to obtain long-term, multi-source traffic data. Traffic operations were quantified across hourly, weekly, and monthly scales, including flow variability, vehicle class composition, axle loads, overload behavior, and speed distributions. Results indicate that the spatiotemporal characteristics of traffic volume show pronounced short-term fluctuations but strong long-term stability. Despite their lower proportion, multi-axle heavy trucks dominate structural loading, with overload ratios exceeding 80% and gross weights approaching 100 t. Over 60% of vehicles operate at medium-to-low speeds (20–60 km/h), extending load duration and increasing pavement damage potential. These combined effects indicate that average indicators alone underestimate true loading demand. The proposed framework provides field-based traffic load spectra and a transferable methodology for traffic monitoring and pavement design optimization across developing regions in Africa.

## 1. Introduction

Highway transportation constitutes a core component of the integrated transport system and plays a fundamental role in supporting economic development and freight mobility. In African countries, the relatively limited development of railways and waterways has resulted in highways carrying the vast majority of passenger and freight traffic [1]. Traffic streams are characterized by a high proportion of heavy-duty trucks, elevated axle loads, and pronounced temporal fluctuations. The combined effects of sustained heavy loading, high ambient temperatures, and constrained maintenance resources markedly accelerate performance degradation of pavements [2,3]. Therefore, accurately characterizing actual traffic operations and loading conditions is an essential prerequisite for pavement structural and material design [4].

Due to historical influences, many African countries have traditionally adopted European technical standards in highway engineering design. However, substantial differences in traffic characteristics often limit the direct applicability of these standards to local contexts [5]. The African region is typically characterized by a large proportion of heavy-duty vehicles, pronounced traffic variability [6]. Pavement service conditions differ markedly from those in countries such as France and the United Kingdom, causing significant challenges to the suitability of existing design methodologies. The lack of systematic field data on traffic conditions has led to design processes that rely heavily on empirical parameters or simplified analogies [7]. Among European technical standards, the French design approach relies heavily on long-term engineering experience and locally calibrated statistical databases [8]. In the absence of representative traffic loading and field performance data, the reliability of parameter extrapolation is inherently limited. By contrast, the British design approach places greater emphasis on material performance classification and stringent construction quality control. Although this strategy can effectively control pavement performance, it imposes higher requirements on material quality, leading increased costs when implemented in African regions [9]. Therefore, accurate characterization of African traffic conditions is essential for ensuring the scientific validity and reliability of design parameters [10]. Conducting systematic monitoring and analysis of traffic characteristics can help address the current lack of representative traffic data. Moreover, it can provide essential data support for establishing pavement design parameters that better reflect the actual traffic conditions in African, thereby improving the reliability of pavement design.

In recent years, the embedded pavement sensors, multi-source sensing technologies, and intelligent data processing have enabled long-term and continuous monitoring of traffic loads [11]. Weigh-in-Motion (WIM) technology is one of the effective technical pathways [12]. This technique enables the continuous acquisition of key traffic parameters without interrupting traffic flow, offering a reliable means to construct realistic traffic load spectra [13]. By deploying WIM systems at representative road sections, vehicle composition, operating speed, and heavy-load characteristics can be monitored continuously and reliably over extended periods [14,15]. Song et al. [16] accurately monitored the pavement traffic loading by integrating WIM with distributed fiber optic sensors. Such datasets provide a foundation for the calibration and verification of pavement design parameters. Zhong et al. [17] developed a traffic input model for the mechanistic–empirical pavement design in Tennessee, USA, based on traffic data collected from nine WIM stations.

However, despite the rapid development of traffic monitoring technologies, most existing studies have focused on developed countries. The traffic composition in these regions differ significantly from those in African countries. Consequently, the traffic load spectra and pavement design parameters derived from these studies may exhibit considerable uncertainty when applied to local highway systems in Africa. Meanwhile, traffic monitoring practices in many African regions primarily serve overload enforcement and administrative management purposes [18]. Research on the coupled effects of temporal traffic variability, vehicle loading characteristics, and operating speed on pavement structural performance remains limited. Therefore, it is necessary to conduct a systematic analysis of key traffic parameters—such as traffic flow characteristics, vehicle composition, axle load distribution, and operating speeds—based on long-term field monitoring data.

Motivated by these needs, this study focuses on a representative highway in Kenya, East Africa, with the objective of establishing a reliable field-based framework for characterizing regional traffic loading conditions. An integrated dynamic traffic monitoring and weighing system was developed to continuously collect high-resolution traffic data. Based on the monitoring dataset, the traffic operational characteristics and loading patterns of typical African highways were systematically analyzed from multiple perspectives, including traffic demand variability, vehicle composition, axle load distribution, overload behavior, and operating speed characteristics. Furthermore, an engineering-oriented traffic parameter database was established to provide reliable field evidence and technical support for pavement structural design and traffic load spectrum development in African regions.

## 2. Methods

### 2.1. Study Area and Research Object

**Figure 1:**
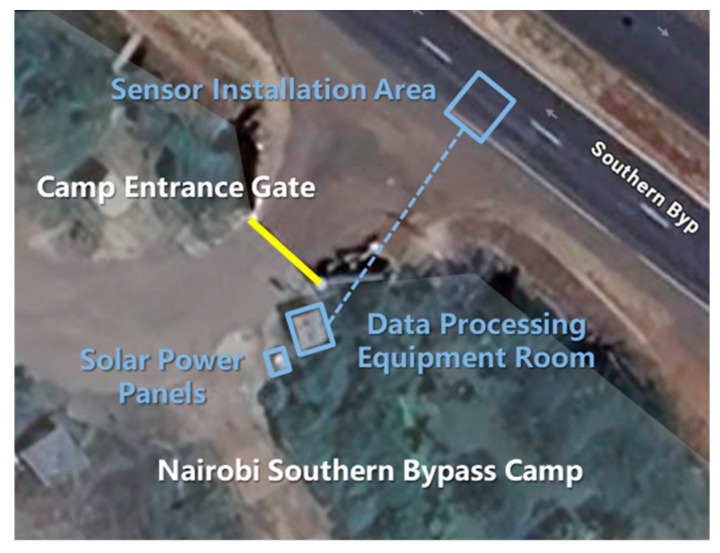
Satellite map of the study area. (The yellow line is the gate and the blue dash line is the cable).

This study focuses on the traffic characteristics of typical highways in the African region, with Kenya selected as a representative case study. The analysis emphasizes vehicle class composition, axle configuration, and traffic operational characteristics. The study section is located on the Bypass Highway in Kenya. The basic information about the study area is shown in Figure 1. This area carries relatively high traffic volumes and exhibits a complex vehicle mix, thereby providing strong regional representativeness.

### 2.2. Deployment of the Integrated Dynamic Traffic Monitoring System

#### 2.2.1. Sensor Components and System Configuration

**Figure 2:**
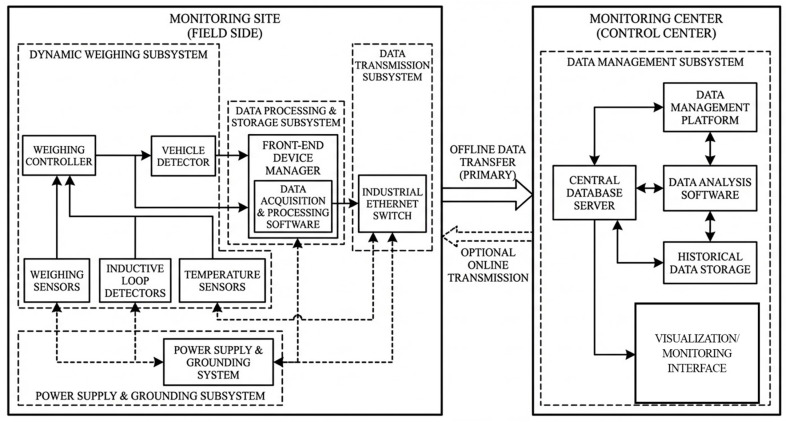
Sensor components.

To enable continuous monitoring of vehicle operating conditions along the study section, an integrated dynamic traffic monitoring system was developed that combines vehicle detection, data acquisition, and on-site processing functions. The system configuration is illustrated in Figure 2.

A piezoelectric quartz WIM sensor (BLS-1, Ascell Sensor, Barcelona, Spain) was used to detect the dynamic stress in real time and convert it into an electrical charge signal. This type of sensor exhibits high sensitivity (piezoelectric constant > 20 pC/N), good output consistency (±7%), and long service life (exceeding 40 million load cycles). The sensors were fixed and sealed using specialized grouting materials to ensure their long-term stability and durability under repeated traffic loading.

To identify vehicles entering and leaving the detection zone, inductive loop detectors (ZCTC-GS-LP, ZHICHENTIANCHI, Beijing, China) were installed in each lane. These detectors identify the presence of vehicles and trigger the weighing process, enabling automatic detection of vehicle passage events. In addition, the loop detectors assist in acquiring key traffic parameters, including vehicle speed, axle spacing, and vehicle classification, which are synchronized with the weighing signals to improve the accuracy of vehicle identification and data acquisition.

The core data acquisition and processing unit of the monitoring system is DCS-P2000. This device adopts a dual-core embedded architecture based on ARM and FPGA, integrating a charge amplifier and a multi-channel data acquisition module. The system provides 64 analog input channels and 24 input–output interfaces, allowing simultaneous access to signals from both piezoelectric sensors and loop detectors. With a sampling frequency of up to 150 kHz, the system meets the requirements of dynamic weighing under high-speed traffic conditions. After charge amplification, filtering, and real-time signal processing, key traffic parameters—including vehicle gross weight, vehicle speed, and vehicle passage time—can be obtained.

The collected vehicle data are transmitted in real time via multiple communication interfaces, including CAN, USB, RS422, RS232, and RS485, and automatically stored in the system database. Each vehicle record is archived as an individual entry containing historical monitoring data. The system also provides real-time monitoring and error logging functions, enabling the identification and recording of equipment status and abnormal data to ensure stable and reliable data acquisition. In addition, the system supports remote online diagnostics and parameter calibration, allowing necessary adjustments to the weighing data.

To ensure long-term stable operation under complex environmental conditions, the data acquisition unit and associated equipment were installed in an outdoor integrated cabinet with lightning protection and surge resistance (IP65 protection level). The monitoring system is powered by solar energy, providing a stable power supply and enabling continuous unattended traffic monitoring.

#### 2.2.2. Sensor Deployment

**Figure 3:**
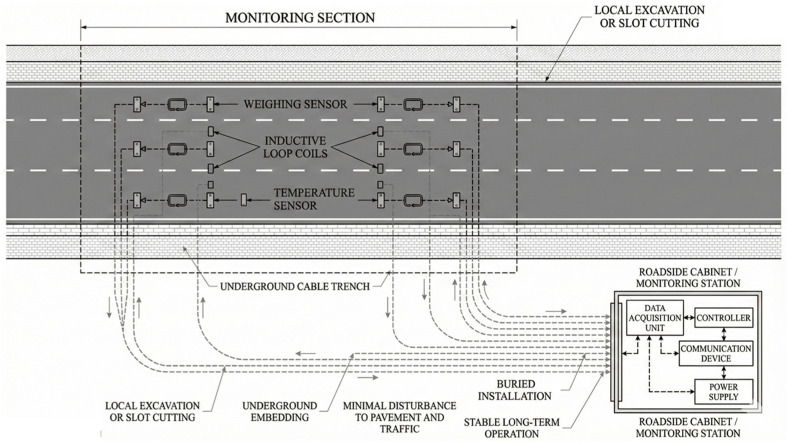
Sensor deployment layout.

To achieve long-term and continuous monitoring of vehicle, the dynamic traffic monitoring system was deployed. Since the system installation involved sensor embedding and cable routing, an on-site survey and preliminary planning were conducted. All cables were installed underground using localized excavation or pavement slot-cutting methods, ensuring stable system operation while minimizing disturbances to the traffic flow. The planned sensor locations and cable routing scheme are illustrated in Figure 3.

#### 2.2.3. System Installation and On-Site Commissioning

**Figure 4:**
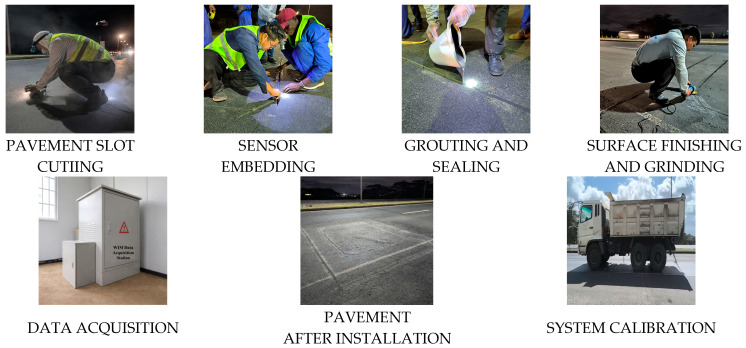
Installation procedure of the sensors.

The installation procedure of the sensors is illustrated in Figure 4. The installation area was cleaned and surface-prepared, and waterproof conduits were pre-embedded for cable protection. After traffic closure, pavement slots were cut, and sensor bases were cast using rapid-hardening materials. The sensor array was then installed, and cables were routed through conduits and connected to the roadside data acquisition and processing unit.

After system parameter configuration, the cut slots were grouted, sealed, and surface-finished to restore normal pavement functionality. After installation, multiple vehicle pass-through tests were performed to calibrate the system and verify the stability and continuity of the monitoring data, ensuring the reliability of vehicle classification and traffic data acquisition.

#### 2.2.4. Data Quality Control and Calibration

To ensure the reliability of WIM monitoring data, a systematic data quality control and calibration procedure was implemented. The data processing mainly included raw data filtering, logical consistency checks of vehicle parameters, and system calibration with drift correction.

First, a preliminary filtering of raw monitoring data was conducted. The WIM continuously records key traffic parameters such as vehicle speed and gross vehicle weight. However, due to factors such as parallel vehicle movement, lane changes, transient sensor signal fluctuations, or communication interruptions, the raw dataset may contain invalid or abnormal records. Therefore, reasonable thresholds based on typical traffic operating conditions were defined to identify and remove abnormal data. Specifically, records with vehicle speeds outside the range of 5–140 km/h, axle loads outside 0.5–20 t, or vehicle lengths outside 2–30 m were considered unrealistic and were excluded from the dataset.

Second, the filtered dataset was subjected to logical consistency checks of vehicle parameters. This step includes axle spacing rationality checks, consistency between vehicle length and axle configuration, and verification of gross vehicle weight against individual axle loads. Records with adjacent axle spacings outside the typical range of 0.8–10 m were removed. In addition, inconsistencies between the sum of individual axle loads and the measured gross vehicle weight were used to identify abnormal records.

Finally, to minimize the influence of long-term sensor performance variations on weighing accuracy, the monitoring system was subjected to periodic calibration and drift correction. Piezoelectric WIM sensors BLS-1 may experience sensitivity changes or zero-point drift due to long-term traffic loading and environmental temperature variations. Therefore, regular system calibration was performed using reference vehicles with known axle loads or standard load data. By comparing the measured axle loads with reference values, the weighing coefficients were adjusted to correct systematic errors. In addition, the system continuously monitored sensor output signals through a remote diagnostic function, and long-term sensor response trends were analyzed using historical monitoring data. Potential sensor drift was then identified and compensated for accordingly. Through the implementation of the above data quality control and calibration procedures, the consistency and stability of the WIM were significantly improved, providing a reliable data foundation for the subsequent analysis of traffic loading characteristics.

### 2.3. Data Analysis Methods

The dataset established covers a five-month observation period from August to December 2025, during which traffic data were continuously collected using a field-deployed monitoring system. This relatively long and uninterrupted monitoring duration ensures that the dataset provides a robust basis for multi-scale traffic characterization.

To improve the statistical reliability and representativeness of the analysis, all available monitoring data within the study period were utilized. In particular, the daily and weekly traffic flow characteristics were derived based on the average values computed from the full dataset, rather than from selected samples or limited observation windows.

#### 2.3.1. Spatiotemporal Characteristics of Traffic Volume

To systematically characterize variations in traffic demand across different temporal scales, the spatiotemporal distribution of traffic volume was statistically analyzed at daily, weekly, and monthly levels. First, based on continuous 24 h traffic monitoring data, hourly traffic volumes were aggregated to construct daily traffic variation curves. Key statistical indicators—including average hourly traffic (AHT), and coefficient of variation (CV)—were calculated using Equations (1) and (2) to quantify the dispersion and fluctuation intensity of traffic demand.

(1) $$AHT=\frac{\sum_{i=1}^{H}Q_{i}}{H}$$

(2) $$CV=\frac{\sigma }{\mu }$$

where *Q_i_* represents the traffic volume during the *i*-th hour (veh/h), and *H* denotes the total number of observation hours (*H* = 24).

In addition, peak-to-valley ratio was calculated using Equation (3) to identify traffic concentration characteristics and to describe temporal load aggregation effects.

(3) $$R_{pv}=\frac{Q_{max}}{Q_{min}}$$

where *R_pv_* represents the peak-to-valley ratio (Peak-to-Valley Ratio), *Q_max_* denotes the maximum traffic volume, and *Q_min_* denotes the minimum traffic volume.

At the weekly scale, the weekly average daily traffic (WADT) and CV were calculated using Equations (2) and (4). Comparisons between weekdays and weekends were conducted to assess the relative influence of commuting and freight activities on traffic demand.

(4) $$WADT=\frac{\sum_{i=1}^{7}V_{i}}{7}$$

where *V_i_* represents the traffic volume on the *i*-th day.

At the monthly scale, the monthly average daily traffic (MADT), CV, and seasonal deviation were calculated using Equations (2), (5) and (6). These indicators were used to evaluate the long-term stability of traffic demand and to distinguish short-term fluctuations from long-term trends, thereby providing multi-scale data support for subsequent traffic load modeling.

(5) $$MADT=\frac{\sum_{i=1}^{n}V_{i}}{n}$$

(6) $$\Delta_{i}=\frac{MADT_{i}-MADT}{MADT}\times 100\%$$

where *n* denotes the number of days in the month, and Δ*_i_* represents the deviation of the *i*-th month from the mean value.

#### 2.3.2. Vehicle Composition and Loading Characteristics

The vehicle classification was performed based on axle configuration in accordance with the Chinese standard GB 1589-2016 [19]. Vehicle composition and loading characteristics were analyzed using single-vehicle records obtained from the WIM system. Vehicles were classified according to axle number and axle configuration to determine the proportion of each vehicle class and overall traffic composition. Gross vehicle weight and individual axle loads were extracted to compare loading levels among different vehicle types. The legal weight limits were determined according to the Chinese standard GB 1589-2016, while vehicles exceeding the legal limit by more than 30% were classified as severely overloaded. In this study, only severely overloaded vehicles were considered in the overloading behavior analysis. These metrics were used to evaluate the intensity of heavy-load operations and the associated potential structural risks to the pavement.

#### 2.3.3. Traffic Operational Characteristics

Vehicle operating speeds were calculated from time differences recorded by inductive loop detectors and were matched with corresponding vehicle class and loading information to enable multi-parameter joint analysis. First, the mean speed, standard deviation, and variability for different vehicle classes were statistically analyzed to assess differences in operating conditions. Speed frequency histograms were then constructed by grouping observations into discrete speed intervals, and the proportion of vehicles within each interval was calculated to characterize overall traffic flow behavior.

Considering the influence of vehicle speed on wheel load duration and the viscoelastic response of asphalt materials, speed data were further analyzed in conjunction with vehicle loading levels to identify sustained loading effects under low-speed, heavy-load conditions. This analysis provides a basis for evaluating the risks of permanent deformation and fatigue damage in pavement structures.

## 3. Results and Discussion

### 3.1. Spatiotemporal Characteristics of Traffic Volume

#### 3.1.1. Daily Traffic Volume

**Figure 5:**
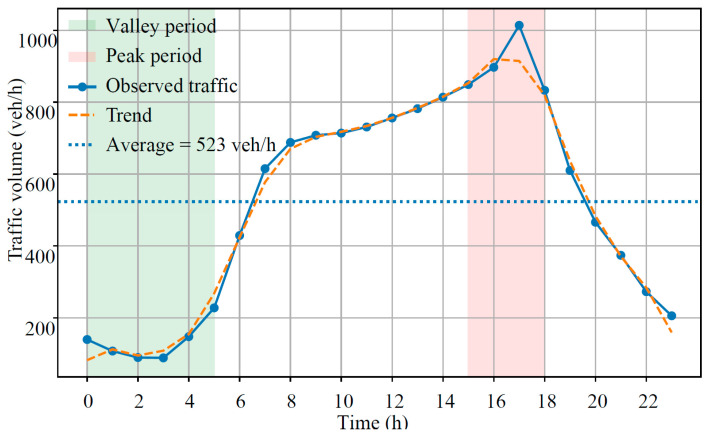
Results of 24 h continuous traffic monitoring.

The calculated daily traffic volume results are presented in Figure 5.

Based on continuous 24 h monitoring data, the temporal distribution of traffic flow on the study section was systematically analyzed. During nighttime hours (00:00–04:00), traffic volumes remained low, ranging from 89 to 148 veh/h, accounting for less than 15% of the peak flow. Traffic demand increased rapidly after 05:00, exceeding 400 veh/h at 06:00 and 600 veh/h at 07:00, and continued rising throughout the day to reach a maximum of 1014 veh/h between 17:00 and 18:00. Traffic volumes gradually declined thereafter and returned to nighttime levels after 21:00.

The daily traffic volume reached 13,362 veh/day, with an average hourly traffic of 557 veh/h. However, the CV was as high as 0.52, indicating substantial temporal dispersion and imbalance. The peak-to-valley ratio reached 11.4, suggesting that traffic loads were highly concentrated rather than uniformly distributed over time. Further analysis showed that cumulative traffic during the four-hour period from 15:00 to 18:00 reached 3593 vehicles, accounting for 26.9% of the daily total, despite representing only 16.7% of the day. This pronounced tidal and clustered traffic pattern is consistent with operational characteristics commonly observed on arterial highways in developing regions, where heavy freight transport, regional logistics activities, and commuter flows overlap, resulting in short-term load amplification.

From a pavement engineering perspective, such non-uniform temporal distributions significantly affect structural performance. Concentrated peak-period loading intensifies load repetitions and accelerates rutting, fatigue cracking, and shear-related damage [20,21]. Reliance solely on average daily traffic (ADT), may therefore underestimate actual loading effects. Accordingly, incorporating time-segmented traffic load spectra or peak amplification factors into pavement design and life prediction is recommended to more accurately reflect service conditions. Targeted maintenance and traffic management during peak periods may further mitigate structural deterioration and improve pavement durability.

#### 3.1.2. Weekly Traffic Volume

**Figure 6:**
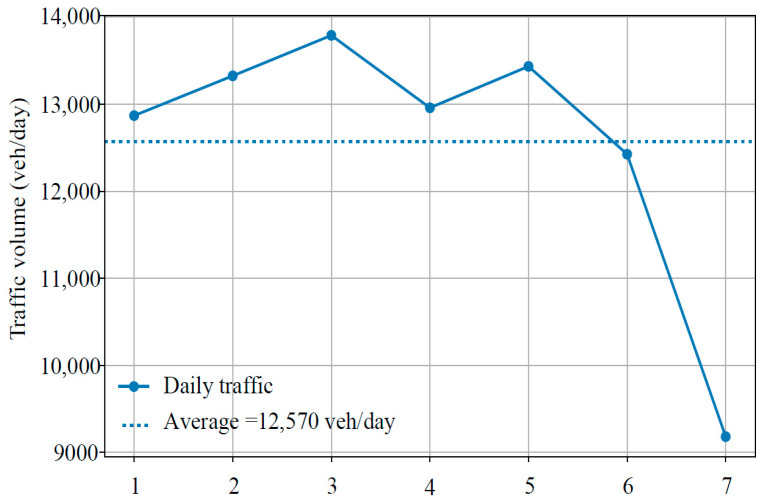
Results of 7-day continuous traffic monitoring.

Weekly-scale statistics further revealed temporal fluctuations in traffic demand, as shown in Figure 6.

Traffic flow exhibited clear weekday–weekend differences. During weekdays (Days 1–5), traffic volumes were highly stable, with an average of 13,276 veh/day and an internal CV of only 3.2%, reflecting consistent commuting and freight activities. In contrast, weekend volumes decreased to 10,804 veh/day, approximately 18.6% lower than weekday levels. These results suggest that traffic demand on the study section is primarily driven by commuting and freight transport rather than leisure travel. From an engineering standpoint, most structural damage accumulates during weekdays, while weekend contributions are relatively minor. Therefore, using weekday average or weighted traffic volumes as representative design inputs may provide a more rational basis for traffic load spectrum development and structural design. Considering weekly variability also improves the accuracy of pavement life prediction and maintenance planning [22].

#### 3.1.3. Monthly Traffic Volume

**Figure 7:**
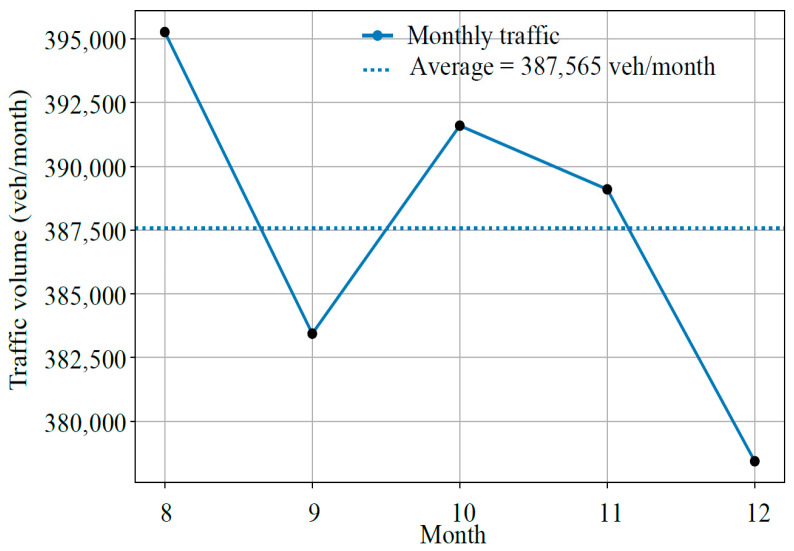
Results of five months of continuous traffic monitoring.

Monthly-scale statistics indicate high long-term stability in traffic demand, as illustrated in Figure 7.

Over the five-month monitoring period, monthly traffic volumes ranged from 378,435 to 395,262 veh/month, with a total traffic volume of approximately 1.94 × 10^6^ vehicles. The corresponding monthly average daily traffic (MADT) was 12,668 veh/day. Statistical analysis showed a CV of 1.66%, indicating minimal fluctuations. Δ_i_ remained within ±2%.

Compared with hourly variability (CV = 52%) and weekly variability (CV = 12.8%), monthly traffic exhibited substantially greater stability, indicating that traffic fluctuations are primarily short-term rather than seasonal. This finding further confirms that the traffic composition is dominated by routine commuting and freight transport rather than seasonal or tourism-related travel. The stability of monthly traffic validates the use of ADT as a representative long-term design parameter, while seasonal correction factors may be simplified or omitted.

### 3.2. Vehicle Composition and Loading Characteristics

**Figure 8:**
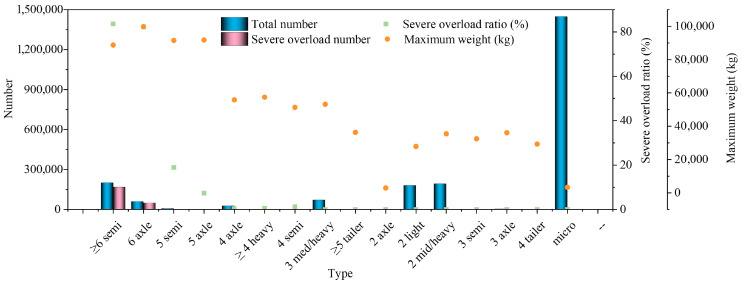
Vehicle class composition and loading characteristics derived from WIM measurements.

The summarized results of vehicle composition and loading characteristics obtained from the monitoring system are presented in Figure 8.

Vehicle classification and WIM data indicate pronounced heterogeneity in traffic composition. During the monitoring period, a total of 2,186,210 vehicles were recorded, of which micro/light-duty vehicles accounted for the largest share (1,446,610 vehicles, 66.2%), followed by two-axle medium–heavy vehicles (8.7%) and two-axle light trucks (8.2%). Although multi-axle heavy trucks represented a relatively small proportion of the total traffic volume, their contribution to pavement loading was disproportionately high.

A total of 216,186 severely overloaded vehicles were identified, corresponding to an overall several overload rate of 9.9%. However, overloading was highly concentrated among multi-axle heavy trucks. The proportion of severe overloads reached 83.61% and 82.29% for semi-trailers and trucks with six or more axles, respectively, indicating critical overloading issues within these vehicle categories. In contrast, vehicles with four or fewer axles exhibited overload rates below 1%, which can be considered negligible. This phenomenon is consistent with data obtained by WIM in Bosnia and Herzegovina through WIM [23]. This pronounced concentration of overloading in multi-axle heavy vehicles may be attributed to several factors. In many developing regions, freight transportation relies heavily on heavy-duty trucks due to limited railway capacity and rapidly growing logistics demand. Transport operators often increase payload per trip to reduce transportation costs and improve operational efficiency, which creates strong economic incentives for overloading. In addition, multi-axle trucks possess greater structural load-bearing capacity and are therefore more frequently used for transporting bulk commodities such as construction materials, minerals, and agricultural products. These operational characteristics make them more prone to severe overloading compared with light-duty vehicles. Furthermore, insufficient enforcement of axle load regulations and limited weigh-station coverage may also contribute to the persistence of overloading behavior in freight transport systems.

The maximum measured gross vehicle showed the severity of heavy loading. Several vehicles exceeded 90–100 t, with six-axle trucks reaching a maximum of 99,800 kg and multi-axle semi-trailers up to 88,760 kg. These values substantially exceeded typical legal limits and may induce excessive pavement stress responses, thereby accelerating structural deterioration. Despite accounting for less than 15% of total traffic volume, heavy-duty trucks dominated cumulative pavement damage due to their extremely high overload ratios. Consequently, overloaded heavy vehicles contribute disproportionately to equivalent single axle loads (ESALs) and constitute the primary source of structural distress. According to the fourth power law of axle load damage, even moderate increases in axle load can lead to exponential growth in pavement damage [24]. Consequently, the presence of severely overloaded multi-axle vehicles can dramatically increase the ESALs and significantly shorten pavement service life. These findings emphasize the importance of incorporating overload correction factors into traffic load spectrum development.

### 3.3. Traffic Operational Characteristics

**Figure 9:**
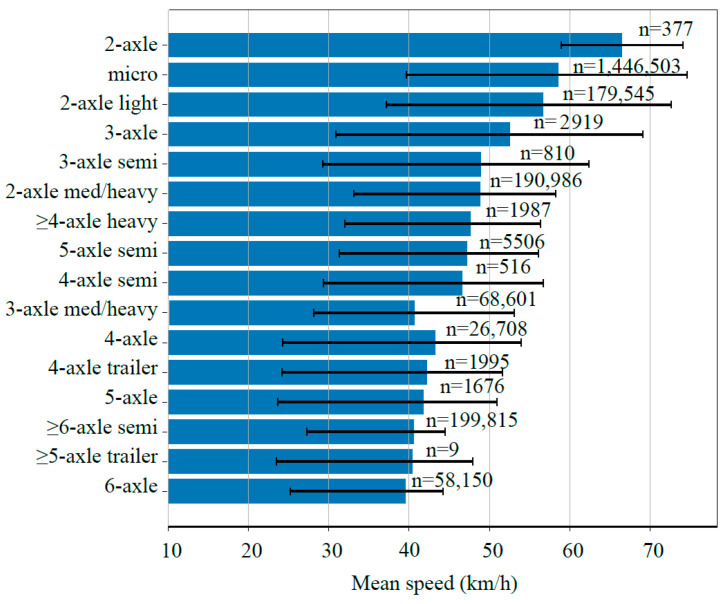
Operating speeds of different vehicle classes.

Based on approximately 1.99 million valid vehicle records, the operating speeds of different vehicle classes were statistically analyzed. The results are presented in Figure 9.

The results indicated significant differences in operational behavior among vehicle types. The weighted average speed of the overall traffic flow was approximately 51.6 km/h, suggesting a moderate operating condition for the study section. Light-duty vehicles traveled considerably faster than heavy-duty vehicles. Micro/light vehicles exhibited the highest mean speed (57.1 km/h), followed by two-axle light trucks (54.9 km/h), whereas heavy multi-axle trucks (six axles or more) operated at substantially lower average speeds of only 34–36 km/h.

Marked differences were also observed in speed variability. Light vehicles showed larger standard deviations (17–19 km/h), reflecting higher maneuverability and frequent speed fluctuations. In contrast, heavy trucks exhibited smaller deviations (8–13 km/h), indicating relatively stable but low-speed operation. This was consistent with their greater mass and limited acceleration capability. The overall speed range was wide, with maximum speeds of light vehicles exceeding 140 km/h and minimum speeds of certain heavy trucks falling below 5 km/h, likely due to overloading, grade resistance, or traffic interference.

Multi-axle heavy trucks—the primary contributors to pavement damage—generally operated at low speeds (typically below 40 km/h). Reduced speeds prolong wheel load duration and intensify the viscoelastic response of asphalt materials. Under the combined effects of high axle loads and frequent overloading, slow-moving heavy vehicles are more prone to inducing permanent deformation and fatigue damage. From a pavement engineering perspective, vehicle speed directly affects load duration and structural response; lower speeds increase load residence time and stress concentration, thereby accelerating rutting and shear-related distresses. Consequently, the “low-speed + overload” condition represents the most unfavorable loading scenario and should be explicitly considered in mechanistic–empirical pavement design and performance prediction. Incorporating speed-dependent traffic load spectra can therefore improve the accuracy of long-term distress forecasting.

**Figure 10:**
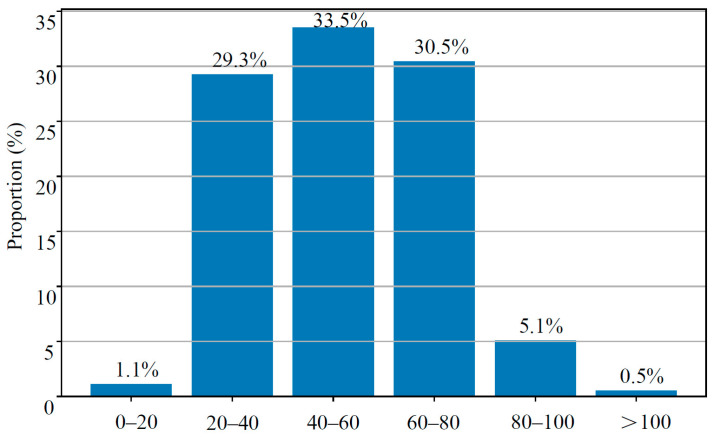
Distribution of vehicle speeds across different intervals.

To further characterize traffic flow conditions, speed frequency distributions were analyzed by grouping samples into six speed intervals, as shown in Figure 10.

Most vehicles operated within moderate speed ranges. The 40–60 km/h interval accounted for the largest share (33.52%), followed by 60–80 km/h (30.46%) and 20–40 km/h (29.26%), with these three intervals collectively exceeding 93% of total traffic. By contrast, high-speed operation above 100 km/h represented only 0.54%, while very low speeds (0–20 km/h) accounted for merely 1.12%.

The predominance of medium-speed operation indicates relatively stable traffic conditions, neither fully free-flowing nor severely congested. The small proportion of high-speed vehicles suggests appropriate geometric design and speed control, whereas the limited presence of very low speeds implies the absence of significant queuing or bottlenecks. This distribution pattern is consistent with typical intercity arterial highways or freight corridors in developing regions, where heavy trucks and routine commuting vehicles dominate.

From a mechanical perspective, vehicle speed governs load duration effects. Approximately 30% of vehicles operated within the 20–40 km/h range, which substantially increases load residence time. When combined with the previously identified high proportion of overloaded heavy trucks, this condition markedly accelerates rutting and permanent deformation. Therefore, the combined “heavy-load + medium/low-speed” scenario is likely the controlling factor for pavement deterioration, and incorporating speed-dependent loading effects is essential for mechanistic–empirical pavement design.

### 3.4. Discussion of Implications of Traffic Loading Characteristics for Pavement Performance

The traffic flow statistics across different temporal scales reveal that the studied highway section exhibits pronounced multi-scale temporal heterogeneity in traffic operations. At the hourly scale, the traffic flow shows a distinct tidal pattern. At the weekly scale, traffic demand presents a relatively stable weekday–weekend pattern. At the monthly scale, traffic fluctuations are minimal. This traffic pattern, characterized by high short-term variability combined with long-term stability, suggests limitations when ADT was used to describe the actual traffic loading process experienced by pavement structures.

Structural damage is closely related to load frequency, load magnitude, and load concentration. Highly concentrated peak traffic flows imply that a large number of repeated loads occur within a short time period, significantly accelerating the fatigue damage accumulation rate of pavement structures. According to damage accumulation theory, such non-uniform load distributions lead to pronounced time-dependent stress–strain responses in pavement structures [25]. Therefore, relying solely on ADT may underestimate the actual structural effects of traffic loading. When developing traffic load spectra for African highways, it is necessary to incorporate time-segmented traffic load functions or peak amplification factors to better represent the influence of peak traffic conditions on pavement performance.

Although light-duty vehicles dominate the traffic volume, structural damage is primarily caused by a relatively small proportion of heavy vehicles. This phenomenon can be explained by the classical fourth power law of axle load damage [26,27]. The law indicates that pavement damage increases exponentially with axle load. In this study, multi-axle heavy vehicles exhibit both a high overloading rate and significantly higher gross vehicle weights than the design standards, resulting in a disproportionately large contribution to ESALs. Consequently, estimating design traffic loads solely based on traffic volume or vehicle counts cannot adequately represent the actual service conditions of the pavement. Instead, probabilistic axle load distributions and vehicle-type weighting factors should be incorporated when constructing realistic traffic load spectra.

Vehicle speed analysis provides further insights into the mechanisms of traffic load application. Vehicle speed not only reflects traffic operating conditions but also directly influences the load duration and viscoelastic response of pavement materials. In asphalt pavement structures, longer load durations lead to more pronounced viscous flow behavior, increasing the likelihood of permanent deformation and shear failure. When low operating speeds are combined with high axle loads and frequent overloading, a significant low-speed–heavy-load coupling effect can occur, intensifying stress concentration and cumulative deformation within pavement structures [28,29]. From the perspective of mechanistic–empirical pavement design, vehicle speed plays a critical role in determining pavement responses. Therefore, incorporating speed-dependent traffic load spectra into traffic loading models can improve the accuracy of pavement response simulations under realistic traffic conditions.

At the broader traffic system level, the traffic operation patterns identified in this study reflect the typical characteristics of trunk highways in many African regions, where regional commuting demands are superimposed with freight transportation activities. This traffic structure often results in highly concentrated traffic loading in both time and space, forming a high-intensity loading environment dominated by heavy freight vehicles. Under such conditions, pavement damage does not accumulate uniformly but is instead concentrated within specific heavy vehicle categories and peak traffic periods. Therefore, pavement management and maintenance strategies should pay greater attention to heavy vehicle regulation and peak-period traffic management. Measures such as strengthening axle load enforcement, optimizing freight logistics operations, and implementing time-based traffic control strategies can effectively reduce the long-term damage caused by overloaded vehicles, thereby extending pavement service life and reducing maintenance costs.

## 4. Conclusions

This study systematically characterized the traffic operations and loading characteristics of representative highways in Kenya using multi-scale traffic monitoring data at hourly, weekly, and monthly levels, combined with analyses of vehicle composition, overload behavior, operating speed, and speed distribution spectra. The main conclusions are summarized as follows:

(1) Traffic operations exhibit pronounced short-term variability but strong long-term stability. This multi-scale temporal heterogeneity indicates that conventional indicators such as ADT cannot adequately capture the real traffic loading process experienced by pavement structures. Therefore, time-segmented traffic load functions or peak amplification factors should be incorporated to better represent actual loading conditions.

(2) Semi-trailers and trucks with six or more axles exhibit severe overload ratios exceeding 80%, with maximum measured gross weights approaching 100 t, far above legal limits. Pavement damage is jointly governed by load magnitude, frequency, and temporal concentration. Highly concentrated traffic during peak periods leads to accelerated fatigue damage accumulation. In the African region, non-uniform load distributions in traffic load modeling should be considered rather than relying on uniform traffic assumptions.

(3) The speed spectrum shows that over 60% of vehicles travel within the 20–60 km/h range, which prolongs wheel load duration and intensifies the viscoelastic deformation response of asphalt layers. The coupling of low speeds, heavy axle loads, and overloading conditions significantly intensifies stress concentration and permanent deformation. Incorporating speed-dependent traffic loading into mechanistic–empirical design frameworks can improve the accuracy of pavement performance prediction.

(4) The identified traffic patterns reflect a freight-dominated loading environment typical of many African highways, characterized by strong temporal and spatial concentration of heavy vehicle loads. Therefore, pavement design and management strategies should emphasize axle load control, freight operation optimization, and peak-period traffic regulation to mitigate long-term damage and extend service life.

Nevertheless, this study primarily focuses on the statistical characterization of traffic features and does not explicitly integrate factors such as climatic conditions, pavement structural properties, and long-term pavement performance data. Future research should expand the monitoring scope to establish a more representative regional traffic loading database. Moreover, incorporating spatiotemporal statistical approaches or machine learning-based predictive models may further enhance the capability to forecast traffic loading variations, thereby improving the accuracy of traffic load analysis and pavement performance prediction.

## Data Availability

Some or all data, models, or code that support the findings of this study are available from the corresponding author upon reasonable request.
